# Three-Dimensional Modeling of Segregation Behavior during Solidification of a Sn-6 wt.% Pb Alloy

**DOI:** 10.3390/ma15041298

**Published:** 2022-02-10

**Authors:** Jian Guan, Zhen-Peng Pu, Si-Cong Zhao, Dong-Rong Liu

**Affiliations:** 1 School of Materials Science and Chemical Engineering, Harbin University of Science and Technology, No. 4 Lin Yuan Road, Harbin 150040, China; jianguan1s@126.com (J.G.); 1920200033@stu.hrbust.edu.cn (Z.-P.P.); zscwr@163.com (S.-C.Z.); 2 Key Laboratory of Advanced Manufacturing and Intelligent Technology, Ministry of Education, School of Mechanical Power, Harbin University of Science and Technology, Harbin 150000, China

**Keywords:** macrosegregation, solidification, three-dimensional simulation, Sn-6 wt.% Pb alloy

## Abstract

In this study, a three-dimensional (3D) solidification model was developed that uses a SOLA algorithm to solve momentum equations and accelerate iterative convergence. The macrosegregation behavior of a sand-cast Sn-6 wt.% Pb alloy was numerically investigated by the developed 3D model. The experiment was carried out for a casting with one side in contact with a graphite chill and the other sides in contact with resin sand. The necessary precision of in-house-developed codes was validated by comparisons with experimentally measured cooling curves and lead concentration distribution. The limitations of the model in fitting experimental results well were discussed. A comparative study between simulations in two-dimensional (2D) and 3D cavities showed that although the general distribution pattern of macrosegregation was slightly affected, the details regarding segregation degree, solute composition distribution over the solidifying domain, solidification time and fluid flow pattern were different. For 2D simulations without boundary walls, the convection behavior was less complicated, and the cooling process was slowed down both in the casting and in the mold.

## 1. Introduction

Macrosegregation has always been an issue of great concern in the casting industry, because its formation directly affects the mechanical properties and the quality of the final product. Some casting defects find their origins in segregation regions [1]. For example, concentrations of stress at certain locations can be caused by a high composition gradient, and cracking is more likely to occur in the solute-segregated area during forging or extrusion of wrought alloys [2]. Moreover, macrosegregation defects usually cannot be eliminated or removed by post-processing techniques, such as heat treatment.

Macrosegregation manifests itself as composition inhomogeneity at the macroscopic scale (casting scale). Several mechanisms have been recognized that could result in macrosegregation [3,4,5,6,7]: (i) thermosolutal convection in liquid and mushy regions; (ii) solidification shrinkage-induced flow; (iii) movement of inclusions; (iv) movement of equiaxed grains and/or dendritic fragments.

For industrial mass production, sand-mold casting is often used [8,9,10], and putting chills at specific locations to accelerate cooling is common. Castings with intricate structures [8] such as engine housings, pump housings and pistons are typically sand-cast [9], sometimes with chills inserted. Owing to their complex geometry and three-dimensional nature, exploring the relationship between processing parameters and macrosegregation based only on experiments is expensive and time-consuming. Therefore, people are more inclined to use numerical simulation methods to study complex physical processes. Reasonable numerical simulation methods can guide experimental research and theoretical analysis and are more intuitive and conducive to data extraction [11,12,13].

Macrosegregation is the phenomenon of uneven composition during solidification, and its distribution can be predicted by numerical simulation. Chen et al. [14] established a two-dimensional (2D) arbitrary Lagrangian–Eulerian model to predict macrosegregation caused by thermal-solutal convection and condensation shrinkage. They further proposed a finite element formula for the “minimal” solidification model to predict macrosegregation during 2D columnar solidification of binary alloys [15]. However, 3D calculations are different from 2D ones, which assume that the effects of lateral walls on fluid flow and heat transfer are negligible [16], resulting in an overestimation of velocity intensity [17]. Therefore, 3D numerical simulations of macrosegregation are necessary and significant both in theory and practice. In recent years, 3D modeling of macrosegregation has become the subject of research efforts.

The majority of the previous 3D numerical studies on macrosegregation formation have mainly focused on directionally solidified castings and on ingots predicted by means of commercial software. Sawada et al. numerically studied the 3D macrosegregation occurrence in the directionally solidified Sn-20 wt.% Bi ingot [18]. Qin and coworkers performed 3D simulations of macrosegregation in the directionally solidified nickel-based superalloy castings [19]. Yuan and Lee numerically coupled the transfer process with the microstructure evolution and predicted the interdendritic segregation in three dimensions [20]. With controlled temperature gradients and pulling velocities, the directional solidification technique aims at producing single crystals or columnar crystals in a specific direction. Compared to directional solidification, the pattern of heat transfer is completely different and may be more complex in sand castings with chills inserted. Alloy melts in contact with sand molds have lower cooling rates than those in contact with chills. An uneven distribution of secondary dendritic arm spacing can exist throughout the casting. As such, all these factors will lead to complicated patterns of fluid flow and macrosegregation. Wu and coworkers developed a four-phase model within the framework of the CFD software package Ansys Fluent and simulated the formation of macrosegregation in a steel ingot with three-dimensional geometries [21]. Li et al. [22] developed a four-phase dendritic model, also within the frame of Fluent, and predicted the macrosegregation, shrinkage cavity and porosity during the solidification of a three-dimensional ingot. Although commercial software is helpful for analyzing macrosegregation, it may have limitations in further model development or extension. We need to establish models by ourselves, integrate algorithms with models, and combine the physical laws existing in the experiments from multiple perspectives, which will make the modelling process more flexible. Therefore, developing a 3D solidification model and the corresponding in-house codes to study the formation of macrosegregation in 3D cavities is necessary [23,24]. Furthermore, the developed model for macrosegregation simulation can be integrated with the cellular automation model [25] or phase field model [26] to predict microstructure formation, which is not common in the literature.

Due to the need to optimize the performance of castings that inevitably solidify under complex conditions, a three-dimensional solidification model was developed in the present work. For the simulation of Sn-6 wt.% Pb sand casting with one graphite chill inserted, an explicit–explicit approach was used to calculate the conservation equations. A temperature-concentration coupling algorithm was created based on an explicit time step. For fluid flow calculations, a SOLA (solution algorithm for transient fluid flow) algorithm with an explicit time step was created to accelerate the iterative convergence. The experiment was carried out, and the simulations were compared with the experimental measurements to validate the numerical implementation of the model. A comparison between two-dimensional simulations and three-dimensional simulations was performed. The effects of mesh size on macrosegregation were studied.

## 2. Model Description

The solidification model describes the conservation of mass, momentum, heat and solute for a binary alloy system undergoing solidification [27]. It can be applied to both two-dimensional and three-dimensional systems. A two-dimensional system is usually a plane of symmetry in the casting; the conservation equations of energy, solute and momentum are solved in two dimensions. A three-dimensional system is where whole casting is studied; the conservation equations of energy, solute and momentum are solved in three dimensions.

Using a volume-averaged technique, the conservation equations were solved using a representative element volume (REV) [28]. To develop the governing equations, some assumptions were made as follows:

(1) Liquid is Newtonian and incompressible.
(2) Natural convection is treated by Boussinesq approximation.
(3) Mold filling is not simulated.
(4) Solid movement is not considered.
(5) Flow induced by solidification shrinkage is not considered.

### 2.1. Conservation of Energy

The enthalpies of the solid and liquid phase are defined as [27]:

(1) $$h_{s}=c_{p}T$$

(2) $$h_{l}=c_{p}T+L_{heat}$$

where $h_{s}$ is the enthalpy of the solid, $c_{p}$ is the specific heat, T is the temperature, $h_{l}$ is the enthalpy of the liquid and $L_{heat}$ is the latent heat.

With the above definitions, the mixture enthalpy can be expressed as:

(3) $$\left[H\right]=f_{s}h_{s}+\left(1-f_{s}\right)h_{l},$$

where $f_{s}$ is the solid fraction and $\left[H\right]$ is the mixture enthalpy.

Therefore, the conservation of energy can be written as [27]:

(4) $$\rho \frac{\partial \left[H\right]}{\partial t}+\rho \nabla \cdot \left(\vec{U}c_{p}T+\vec{U}L_{heat}\right)=\lambda \nabla \cdot \left(\nabla T\right)$$

where ρ is the density, t is the time, $\vec{U}$ is the flow vector and λ is the thermal conductivity. $\vec{U}$ is obtained by solving the following momentum conservation equation and consists of three components of u, v and w.

### 2.2. Conservation of Solute

The mixture concentration is written as:

(5) $$C_{mix}=f_{s}C_{s}+\left(1-f_{s}\right)C_{l}$$

where $C_{mix}$ is the mixture concentration and $C_{l}$ is the liquid concentration. $C_{s}$ is the macroscopic solid concentration treated by performing volume-averaging over the solid phase in REV. Neglecting the dispersive and macroscopic diffusion fluxes, the solute is transported by fluid advection through the domain. We can write the conservation of solute as [27]:

(6) $$\rho \frac{\partial C_{mix}}{\partial t}+\rho \nabla \cdot \left(\vec{U}C_{l}\right)=0$$

where $\vec{U}$ is obtained by solving the following momentum conservation equation and consists of three components u, v and w.

On the basis of the assumption of a linear phase diagram, the liquid concentration and temperature are related through the liquidus line:

(7) $$T=T_{m}+m_{l}C_{l}$$

where $T_{m}$ is the melting temperature and $m_{l}$ is the liquidus slope.

### 2.3. Conservation of Mass and Momentum

The mass conservation equation is [27]:

(8) $$\rho \nabla \cdot \left(\vec{U}\right)=0$$

The X-direction momentum equation is:

(9) $$\rho \frac{\partial u}{\partial t}+\rho \nabla \cdot \left(\vec{U}u\right)=-\frac{\partial P}{\partial x}-\frac{\mu_{l}u}{K_{p}}+\mu_{l}\nabla \cdot \left(\nabla u\right)$$

The Y-direction momentum:

(10) $$\rho \frac{\partial v}{\partial t}+\rho \nabla \cdot \left(\vec{U}v\right)=-\frac{\partial P}{\partial y}-\frac{\mu_{l}v}{K_{p}}+\mu_{l}\nabla \cdot \left(\nabla v\right)$$

The Z-direction momentum:

(11) $$\rho \frac{\partial w}{\partial t}+\rho \nabla \cdot \left(\vec{U}w\right)=\;-\frac{\partial P}{\partial z}-\frac{\mu_{l}w}{K_{p}}+\mu_{l}\nabla \cdot \left(\nabla w\right)+\rho \vec{g}\left[\beta_{T}\left(T_{ref}-T\right)+\beta_{C}\left(C_{ref}-C_{l}\right)\right]$$

where u is the value of the direction X component of $\vec{U}$, v is the value of the direction Y component of $\vec{U}$, w is the value of the direction Z component of $\vec{U}$, $\mu_{l}$ is the dynamic viscosity, $K_{p}$ is the permeability in the mush, P is the pressure, $\beta_{T}$ is the thermal expansion coefficient, $\beta_{C}$ is the solutal expansion coefficient, $\vec{g}$ is the gravity vector, $T_{ref}$ is the reference temperature and $C_{ref}$ is the reference concentration equal to initial concentration.

The variable $K_{p\;}$ is termed as the mushy permeability. Here an isotropic model is adopted:

(12) $$K_{p}=\frac{d_{s}^{2}}{180}\left[\frac{{\left(1-f_{s}\right)}^{3}}{f_{s}{}^{2}}\right]$$

where $d_{s}$ is the equivalent grain size.

### 2.4. Numerical Details

The form of discretized energy and concentration equations using an explicit time step scheme and finite difference method was similar to that described in Ref. [27]. A staggered Cartesian mesh with uniform spaced grids was used. Variables are defined at the center making the discrete flux-conservative equations. Diffusion terms were discretized with a standard second-order finite difference procedure. Velocity components were evaluated on the control volume interfaces. The SOLA technique was applied. The values of pressure and flow velocity were adjusted in an iteration process until the mass conservation equation was satisfied [29]. The convergence criterion for the iteration loop was when the residual mass source within each control volume fell below 1 × 10^−5^. The solidus temperature was equal to the eutectic temperature in the present model. The solid fraction increasing to one was the criterion used to finish solidification. The time step was determined by the maximum velocity at the previous time step. The mesh size was 4 × 4 × 4 mm^3^, which is fine enough to capture all of the fundamental transport phenomena, while allowing for reasonable computational costs.

## 3. Experiment

**Figure 1:**
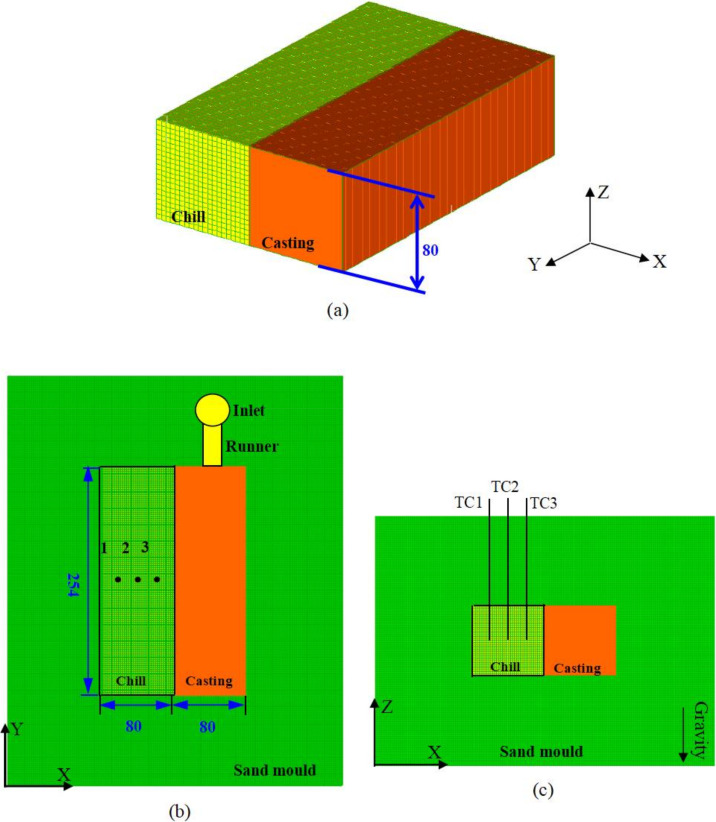
Schematic of the experimental setup: (**a**) three-dimensional representation of the side-cooled experiment; (**b**) two-dimensional top view; (**c**) two-dimensional front view. Thermocouples (TC1, TC2 and TC3) were positioned in a graphite chill. All dimensions are in millimeters.

The casting experiment was performed on a one side-chilled rectangular cavity. The model alloy chosen was Sn-6 wt.% Pb for three reasons: (i) it exhibits a stronger tendency for macrosegregation due to a small partition coefficient; (ii) its low melting point, which makes it suitable for laboratory investigations; (iii) the availability of its thermophysical properties and phase diagram data for simulations. For the present experimental design, some ideas were borrowed from Ref. [30], with the aim of inducing macrosegregation in a small-sized sample. A schematic of the experimental set-up is shown in Figure 1.

A graphite chill was placed at one cavity/mold interface with an area of 80 × 254 mm^2^. Except the chilled face, all faces were in contact with resin sand. The thickness of the sand around the casting and chill was 80 mm. Three K-type (NiCr-NiSi) thermocouples, TC1, TC2 and TC3, were positioned in the central plane of the chill; they had the same inserting depth of 40 mm. The distance between each was about 20 mm. The relative accuracy of the temperature measurement was ±1 °C.

**Figure 2:**
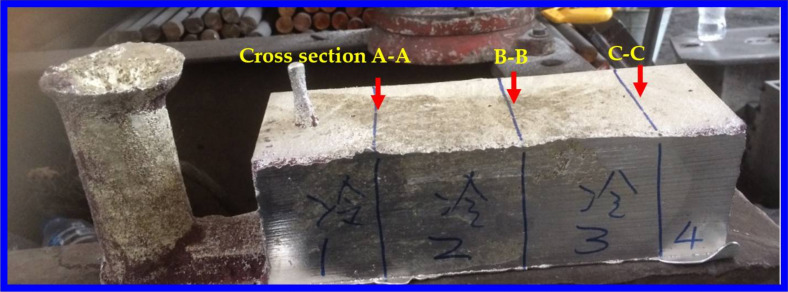
Photograph of solidified Sn-6 wt.% Pb alloy casting with three cross sections for cutting.

Molten Sn-6 wt.% Pb was prepared in a resistance furnace. Both the sand mold and the graphite chill were preheated to 70 °C for drying purposes before pouring. The pouring temperature was about 280 °C, with the melt superheating at approximately 55.0 °C. The total filling time was about 30 s. As shown in Figure 2, the solidified casting was sectioned at three positions along the A–A, B–B and C–C planes. All of the three planes were macroetched to reveal the grain structures. Only the central plane B–B was used to measure the lead concentration.

A standard metallurgical technique was used for the observation of grain structures. The specimens were first polished using sandpaper, and then etched for about 90 s in a mixture of 75% Vol HCl and 25% Vol HNO_3_ [31]. After chemical attack, the specimens were washed with absolute ethyl alcohol and dried with a blowing machine. The grain structures were photographed. A chemical analysis was used to measure the lead concentrations. One side of the B–B plane was used for macrostructure observation and the other side was divided into 6 (X) × 6 (Z) segments. At the center of each segment, an 8.0 mm-diameter drill was used to obtain metallic fragments, with a penetration depth of 10.0 mm. The estimated relative uncertainty in the concentration measurement was ±0.2 wt.% (relative to the nominal concentration).

## 4. Results and Discussion

The 3D simulations of thermal, concentration and flow fields were performed for the casting without considering the pouring system (Figure 1a). The thermophysical properties of the Sn-6 wt.% Pb alloy and the modeling parameters are listed in Table 1 and Table 2, respectively. Here it was assumed that all thermo-physical parameters are constant. For the calculation of momentum transfer in the mushy zone, the SDAS (secondary dendrite arm spacing) was an input parameter for estimating the permeability (see Equation (12)). In Ref. [32], by controlling the temperatures at the two end sides, a constant horizontal temperature gradient of 0.4 °C mm^−1^ and cooling rate of 0.03 °C s^−1^ were set up. The SDAS was measured in the range of 86–94 μm through the as-cast sample of Sn-6.5 wt.% Pb alloy. In the present experiment, the temperature gradient along the X direction changed with time during the solidification, and the cooling rate of each point was not constant. During the solidification, the temperature gradient between the casting surfaces along the X direction decreased from 2.0 °C mm^−1^ to 0.4 °C mm^−1^. The SDAS was expected to vary with position and time. To simplify this, here the characteristic SDAS was set to 100 μm, as used in Ref. [32]. The solutal expansion coefficient was calculated by means of the linear interpolation of the data given in [32].

### 4.1. Comparison of Simulation with Experiment

In the simulation, the temperatures of the graphite chill and sand mold were set at 25 °C and the pouring temperature at 280 °C. Table 3 lists the measured lead concentrations for 6 × 6 grids. We verified that the average concentration of all the measured points was close to the nominal concentration, 6.011 wt.% Pb, for the Sn-6 wt.% Pb alloy.

**Figure 3:**
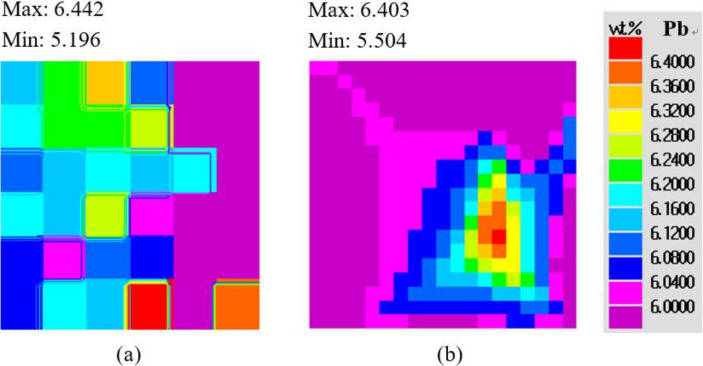
Comparison of Pb concentration on the central B–B plane: (**a)** experimental measurement, (**b**) simulation.

Figure 3 shows a comparison of the predicted and measured distributions of lead concentration. The numerical result is consistent with the experiment in two aspects: the lower part of the cavity was characterized by a higher degree of positive segregation; the area with the negative segregation appeared along the right mold wall. The density of lead is higher than that of tin, leading to the accumulation of heavier lead-rich liquid at the bottom. The relatively lower heat transfer rate between the alloy and the right sand mold led to the occurrence of a negative segregation band. There is one noticeable discrepancy—the positive segregation near the metal/chill interface in the experiment (Figure 3a) was not reproduced in the present 3D simulation (Figure 3b). Since the development of a 3D model is based on some assumptions (Section 2), the influence of each assumption on this discrepancy was analyzed.

Since the normal gravity casting process (without an external force field) was studied, the fluid flow was in a natural flow state. Thus, assumptions (1) and (2) were reasonable, and natural convection was a driving force for the formation of macrosegregation.

For assumption (3), since the mold filling was not simulated, the initial temperature distribution in the casting was affected. In the experiment, the initial temperature distribution was uneven and was lower than the pouring temperature, because the heat transfer occurred in the melt and between the melt and mold materials during the filling process. In the present simulation, the initial temperature distribution in the casting was uniform and was equal to the pouring temperature. The limitation of this assumption is that there is a difference between the simulated temperature field and the experimental one. This difference may contribute to the discrepancy in the prediction of macrosegregation.

For assumption (4), the solid movement was not considered, which may have had an influence on the simulated segregation distribution. After nucleation, the formed equiaxed crystals have different concentrations and densities. Two-phase flow, the free motion of equiaxed crystals and fluid flow, will make the concentration distribution in the casting more complicated.

For assumption (5), the shrinkage-induced flow along the fast-cooled direction was not modeled, which is considered a major reason for the difference noticed between the simulated and the experimental segregation distributions. At the initial stage of solidification, the high-temperature melt touches the low-temperature graphite chill. Then, a fast solidification occurs that corresponds to a large degree of solidification shrinkage. A gap with negative pressure is generated between the columnar dendrites due to shrinkage. The shrinkage-induced flow could draw the lead-rich liquid from the inner region toward the chilled surface [33]. As the shrinkage-induced flow was not simulated, the positive segregation at the surface region was not reproduced.

Moreover, since the experimental measurements and the numerical calculations were performed on grids with various densities, it is noted that Figure 3a,b have different numbers of squares. In the present study, the mesh dimension used in the experimental measurement was about 13 × 13 mm^2^, whereas the mesh for simulation was 4 × 4 mm^2^.

**Figure 4:**
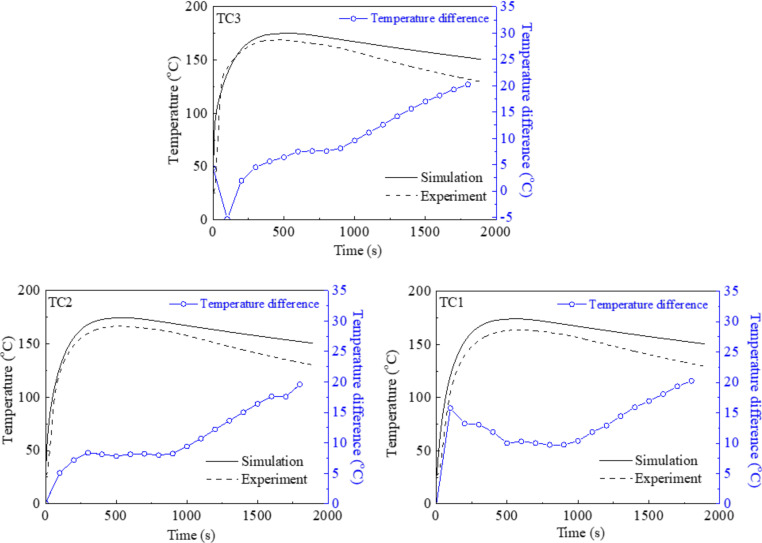
Comparison of cooling curves at TC1, TC2 and TC3. Temperature differences between simulations and experiments are superimposed. Thermocouples were inserted in the graphite chill.

Figure 4 compares the predicted and experimental cooling curves at three thermocouples (TC1, TC2 and TC3) located in the graphite chill. The predicted cooling curves were in fair agreement with those obtained from the experiments. The simulated curves were above the experimental ones, suggesting that the predicted cooling rate is lower. The temperature difference between the simulated and experimental curves was smaller (6 °C–10 °C) during the heating process, and got larger with the elapse of time. For example, for TC3, the temperatures were 174 °C (sim) and 168 °C (exp) at 390 s, and the temperatures were 152 °C (sim) and 131 °C (exp) at 1800 s. Three reasons may explain this discrepancy in the cooling curves. The constant heat conductivity was used in the simulation; this parameter usually increases with decreasing temperature. The heat transfer coefficients at the alloy/chill and alloy/sand interfaces were selected as close values, but were not an exact duplication of the experiment, and were also used as constants in the simulation. They could be more precisely deduced by the inverse algorithm and were expressed as a function of solidification time [34]. The mold-filling process, as mentioned above, was not simulated.

The Sn-Pb has always been used in the study of segregation. In the last five years, the research on numerical simulations of the segregation of Sn-Pb alloys is mainly based on two-dimensional (2D) models. Kumar et al. [35] developed a 2D model of mushy zone instability for the characterization and prediction of channel segregation in Sn-5 wt.% Pb alloy casting under horizontally solidified conditions. Wu et al. [36,37] established a 2D model to simulate the complexity of transient flow and macrosegregation distribution in a Sn-Pb alloy under vertically solidified conditions. Wu et al. [38] further developed a 3D model to study the formation of the as-cast structure and macrosegregation of Sn-10 wt.% Pb alloy under horizontally solidified conditions. In the above-mentioned research, the heat transfer direction was strictly controlled, and so could be considered directional solidification. In addition, as there was no chilling from the mold surface, the shrinkage-induced flow was not considered.

In the present research, although directional solidification was not achieved in a strict manner, the simulated tendency of macrosegregation, negative segregation in the area close to the graphite chill, and positive segregation in the central-lower region was consistent with the simulated results above.

### 4.2. Comparison of 2D and 3D Simulations

**Figure 5:**
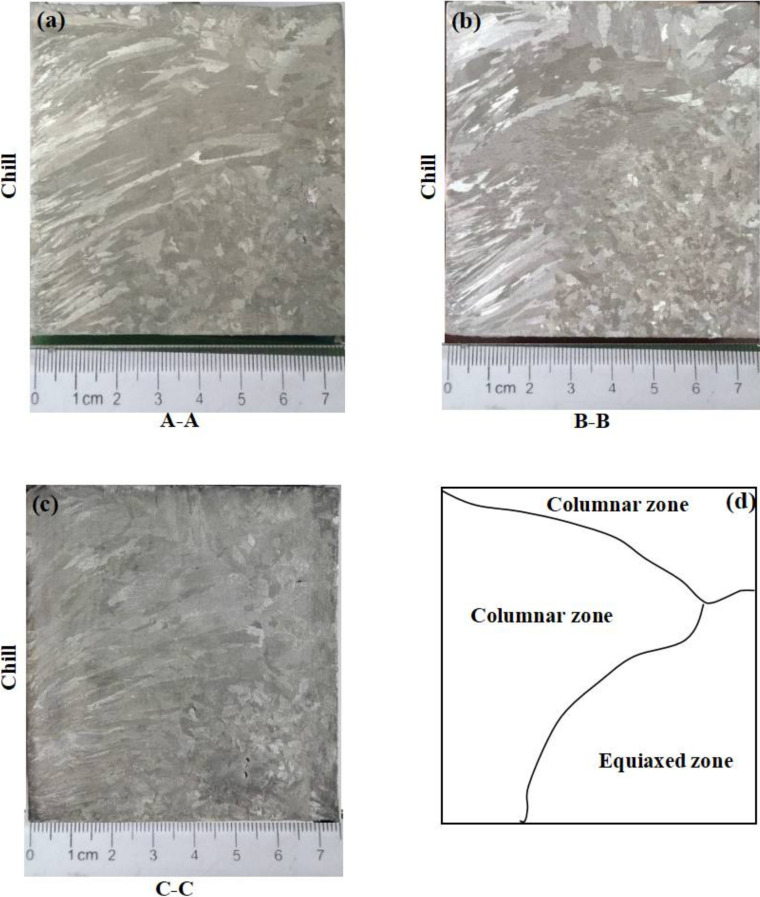
Macrostructure of Sn-6 wt.% Pb alloy on cross sections: (**a**) A–A, (**b**) B–B and (**c**) C–C and (**d**) schematic of grain structure zones. Plane B–B is the central plane.

Figure 5 presents the grain structures at three planes. The grain structures were similar. Since the graphite chill exhibited a higher heat extraction rate, columnar grains that nucleated on the chill surface were formed. Due to the lower chilling efficiency of the sand mold, short columnar grains initiated from the top surface. The equiaxed zone was formed at the lower right. It seems that the solidification of each plane was not affected very much by the neighboring plane. Therefore, the two-dimensional and three-dimensional simulations should be compared to check whether it is reasonable to assume a two-dimensional solidification behavior along the plane normal to the chill.

**Figure 6:**
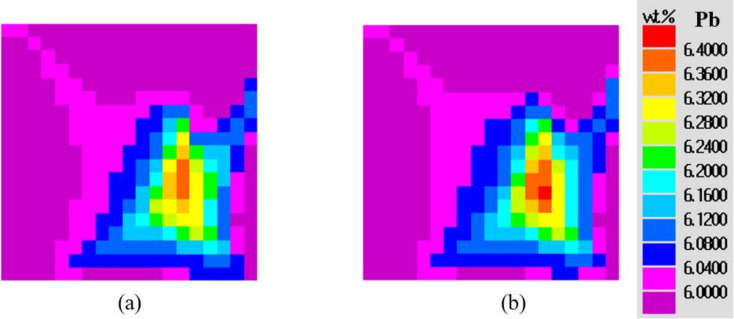
Comparison of Pb concentration on the central B–B plane after solidification: (**a**) 2D simulation at 2000 s, (**b**) 3D simulation at 1050 s.

Figure 6 shows the maps of the concentration field calculated by the two-dimensional model and three-dimensional model, respectively. The distribution patterns of the lead concentration in the 2D and 3D cases were similar. However, the details regarding the segregation were different. The ratio of the positive-segregation area to negative-segregation area was 0.87 (2D) and 0.95 (3D), respectively. For the 2D model, the calculated maximum and minimum lead concentration was 6.39 wt.% and 5.57 wt.%, respectively. For the 3D case, the calculated maximum and minimum was 6.403 wt.% and 5.504 wt.%, respectively. The macrosegregation degree in the central plane was increased in the 3D simulation.

**Figure 7:**
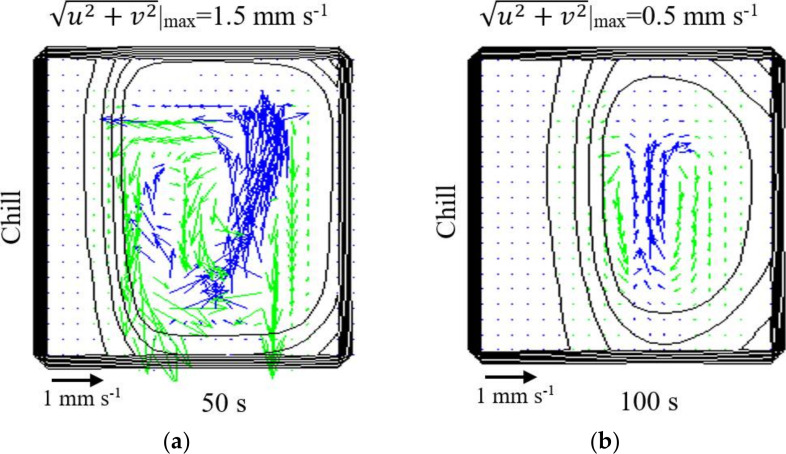
Two-dimensional simulation of flow vectors at (**a**) 50 s and (**b**) 100 s. The superimposed solid black lines are solid fraction contours. The upward flow is blue and the downward flow is green.

**Figure 8:**
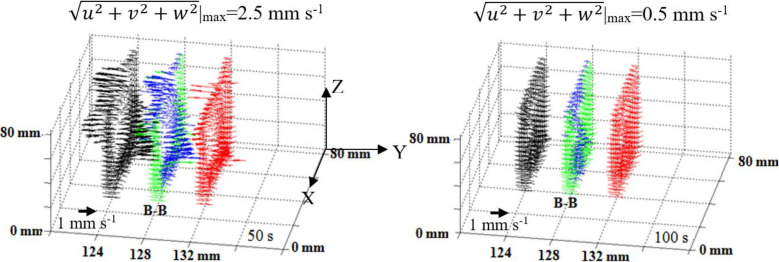
Three-dimensional simulation of flow vectors at 50 s and 100 s. Only vectors on the central plane B–B (upward flow is blue and downward flow is green) and its two neighboring planes (black color and red color) are displayed.

Figure 7 and Figure 8 show the flow fields in the form of vectors for the 2D and 3D models, respectively. If the velocity component along the Z axis is in the same direction as gravity, the corresponding flow vector is displayed in a green color to show a downward flow. Otherwise, the flow vector is displayed in a blue color, indicating an upward flow. The flow patterns in the two models were close. The cooling from the left and the right walls induced thermal buoyancy forces that established a downward flow ahead of the solidification front. With the development of the mushy zone, the formation of the solid was accompanied by the enrichment of the interdendritic liquid with lead. As Pb is heavier than Sn, this enrichment served to increase the density of the liquid. Therefore, the solutal buoyancy forces enhanced the thermal buoyancy forces, driven by the temperature gradients, and also favored a downward flow. In Figure 7a, in the inner portions of casting, the flow was unstable; counter-clockwise flow cells coexisted with clockwise cells. Before the superheat was totally dissipated, a relatively steep temperature gradient was formed between the solid zone and the liquid zone. The enriched liquid, which tended to sink, encountered the superheated liquid, which tends to float, making the flow pattern more complicated. In Figure 7b, two well-organized flow cells in different directions are set up. A downward flow ahead of solidification front and an upward flow in the central liquid was accomplished as the temperature gradient between the solid and the mush became flat, at 0.9 °C mm^−1^.

In Figure 8, besides plane B–B, its two neighboring planes are taken into account, each consisting of one layer of mesh. Flow vectors are displayed in black and red. The fluid flow along the Y axis is clear. As such, there is a solute exchange between the different planes vertical to the Y axis. This three-dimensional convection finally leads to the uneven distribution of lead concentration on the different planes/surfaces.

**Figure 9:**
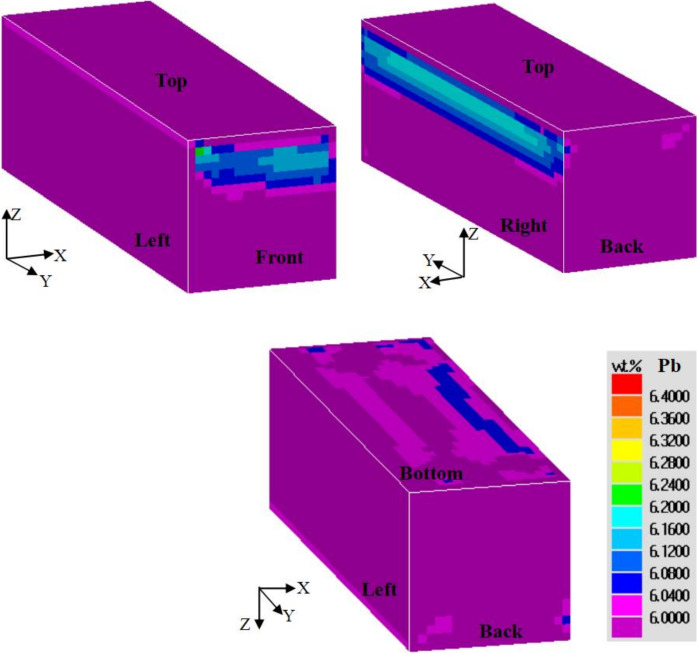
Distributions of lead concentration on the surfaces of the casting observed from different view angles. The left surface is in contact with the chill.

**Figure 10:**
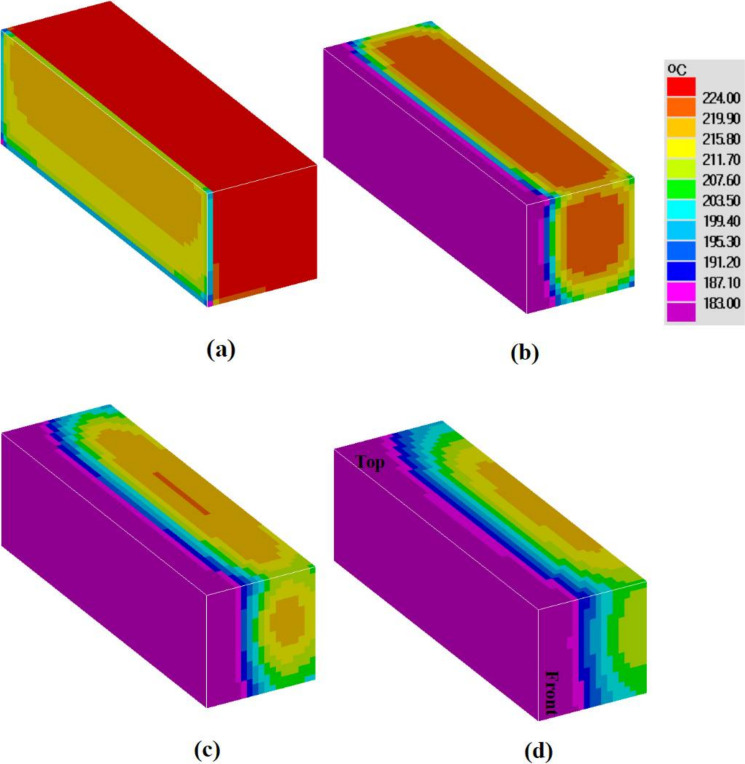
Time evolution of temperature fields at: (**a**) 5 s, (**b**) 50 s, (**c**) 150 s, (**d**) 250 s.

The distributions of Pb concentration on the six surfaces of the casting are shown in Figure 9. The lead concentration presented with an asymmetrical distribution on the back and front surfaces. This was attributed to the asymmetrical flow field in the three dimensions. The degree of positive segregation was higher on the bottom surface compared to that on the top surface, as lead has a higher density than tin. As shown in Figure 10, the left and the right surfaces have different cooling rates. Since the cooling rate and the final solute concentration are related, the right surface in contact with the sand mold, with a lower cooling rate and a longer fluid flow time, finally showed a larger area undergoing positive segregation.

**Figure 11:**
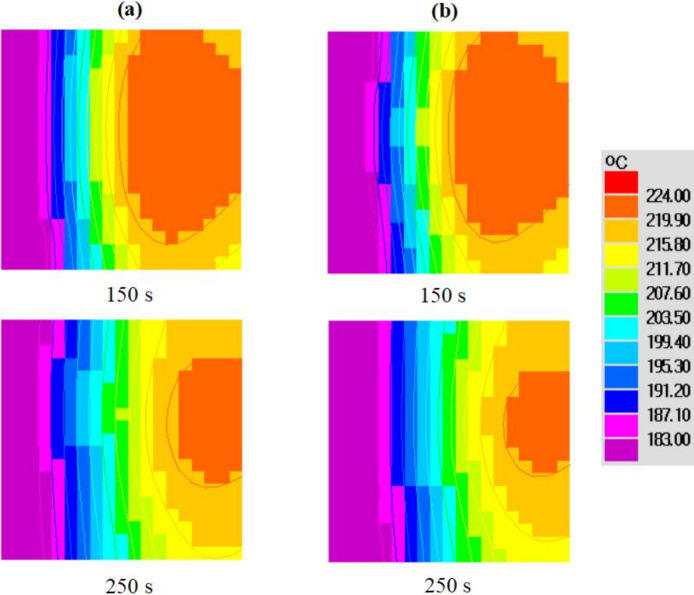
Comparison of temperature fields between the (**a**) 2D simulation and (**b**) 3D simulation.

**Figure 12:**
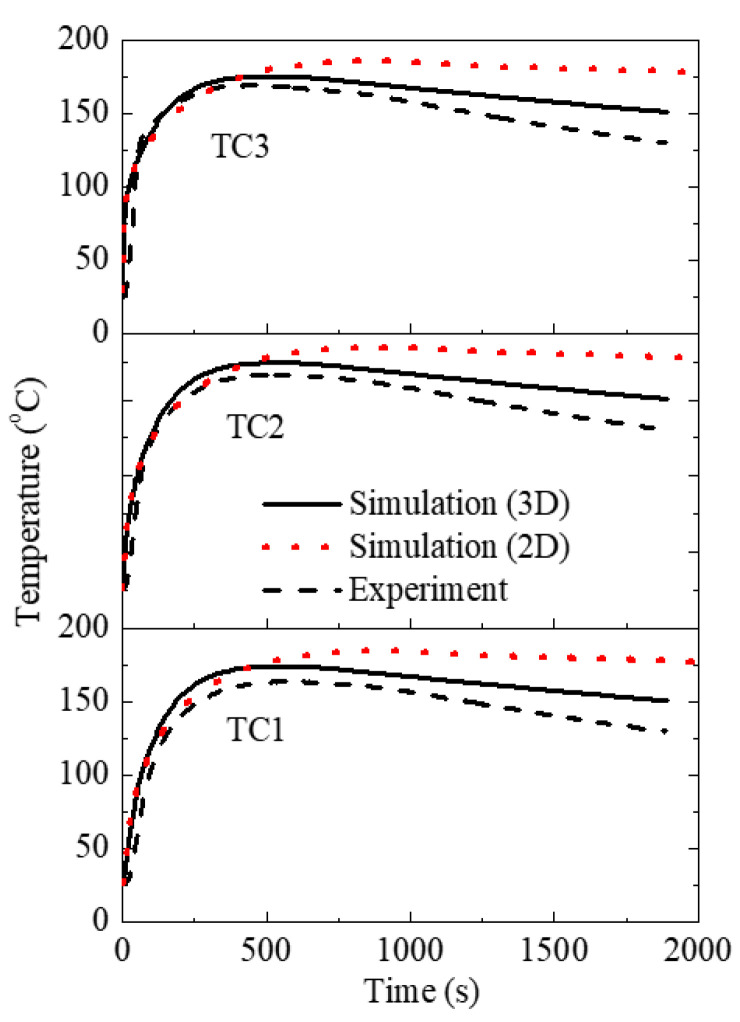
Predicted cooling curves by 2D and 3D models. TC1–TC3 have the same temperature scale.

Moreover, in Figure 10, the isotherms on the top surface look like the letter “C” at 150 s and 250 s; there is an obvious heat transfer from the casting to the sand mold through the back and front surfaces. On the front surface, the isotherms were more distorted at the bottom region, due to the heat conductivity between the alloy and the sand mold, together with the heat transfer caused by the downward flow. For two-dimensional simulations, it is usually assumed that the central plane is not influenced by other parallel walls. In Figure 11, we can see that the 2D simulation predicted a lower cooling rate. The total solidification time was 2000 s and 1050 s for the 2D and 3D simulations, respectively. Furthermore, as shown in Figure 12, there was a larger difference in cooling curves between the 2D model prediction and the experimental measurement. Compared to the 3D model, the heat transfer was “slowed down” in the 2D model, with the assumption of heat insulation along one co-ordinate direction.

**Figure 13:**
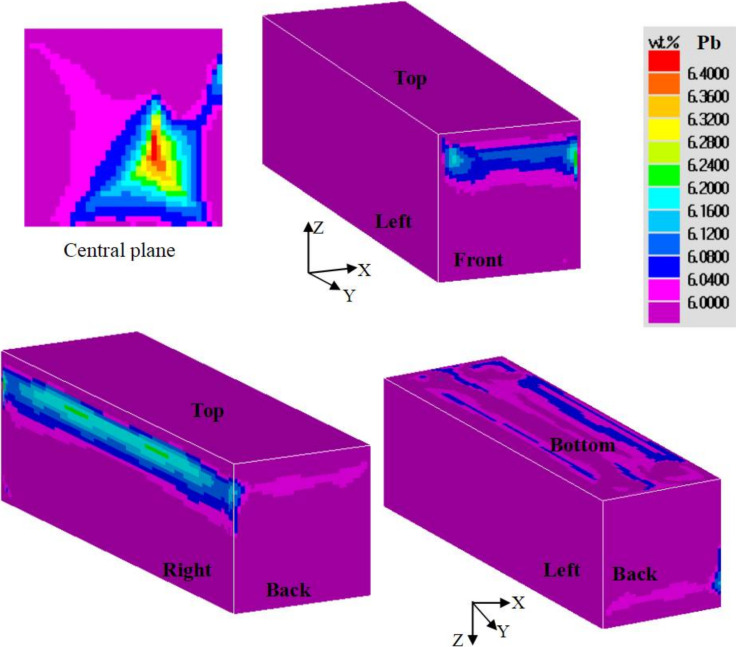
Final distribution of Pb concentration in the casting simulated with a finer grid size of 2 × 2 × 2 mm^3^.

The mesh size was refined to 0.2 × 0.2 × 0.2 cm^3^ and the simulated lead concentration was displayed in Figure 13. Reconsidering Figure 6b and Figure 9 (0.4 × 0.4 × 0.4 cm^3^), we can see that the general macrosegregation pattern was not modified by finer meshes, although some local details were different—for example, on the bottom surface. This is because refining grids did not significantly affect the calculation of the conservation equations. Since the mesh size of 4 × 4 × 4 mm^3^ or 2 × 2 × 2 mm^3^ was larger than the chosen secondary dendrite arm spacing (100 μm), the requirement that REV should be small with respect to the extent of the mushy region, but containing a number of arm spaces, was satisfied [39]. The low sensitivity of the predicted macrosegregation to the mesh size is consistent with the numerical investigations in Ref. [40]. Compared to macrosegregation, the formation of mesosegregation is more sensitive to meshes. Ref. [40] stressed that only a fine mesh (about 0.03 mm) was sufficient to describe the formation of channel segregates (mesosegregation), since the spatial discretization with a coarse mesh gave inadequate accuracy for the determination of transport within channels. In the present work, the cooling rate created in the casting was rapid. The established solidification conditions did not favor the occurrence of solute channels.

## 5. Conclusions

(1) The macrosegregation behavior of a sand-cast Sn-6 wt.% Pb alloy with one graphite chill inserted into the mold was numerically investigated by solving three-dimensional conservation equations. The simulated cooling curves showed reasonable agreement with the experimentally measured curves extracted from the chill. There were differences between the measured and predicted lead concentration fields, since the fluid flow induced by shrinkage was not calculated. However, as the trends for the concentration distribution were similar, it was verified that the present 3D model is capable of predicting some reasonable macrosegregation patterns.

(2) The two-dimensional simulation showed a macrosegregation pattern close to the three-dimensional calculation. However, the solidification rate and segregation degree in the 2D model were lower than those in 3D model. The fluid flow was more complicated in 3D simulation and gave rise to appreciable variation of the solute composition over the entire solidifying domain.

(3) Changing the mesh size from 4 × 4 × 4 mm^3^ to 2 × 2 × 2 mm^3^ did not significantly modify the results, because the selected REV length scale was consistent with the assumptions used to build the model.

The developed 3D model still needs to be improved by considering the variations of thermophysical parameters with temperature and other physical factors affecting fluid flow in future work.

## Figures and Tables

**Table 1:** Thermophysical properties of Sn-6 wt.% Pb, numerical parameters used in present simulations.

Parameter	Value
Melting temperature (°C)	232.0
Liquidus temperature (°C)	224.3
Eutectic temperature (°C)	183.0
Partition coefficient	0.0656
Liquidus slope (°C wt.%^−1^)	−1.286
Eutectic composition (wt.%)	38.1
Initial mass fraction (wt.%)	6.0
Thermal expansion coefficient (°C^−1^)	6 × 10^−4^
Solutal expansion coefficient (wt.%^−1^)	−0.0032
Dynamic viscosity of liquid phase (kg m^−1^ s^−1^)	0.002
Liquid density (kg m^−3^)	7250.0
Latent heat (J kg^−1^)	6.07 × 10^4^
Specific heat (J kg^−1^ °C^−1^)	242.0
Thermal conductivity (W m^−1^ °C^−1^)	33.0
Secondary dendrite arm spacing (µm)	100.0
Mesh size (cm^3^)	0.4 × 0.4 × 0.4
	0.2 × 0.2 × 0.2

**Table 2:** The boundary conditions used in the current simulations.

	Parameter	Value
Heat transfer coefficient	Interface casting/chill (W m^−2^ °C^−1^)	4500.0
	Interface casting/sand (W m^−2^ °C^−1^)	500.0
	Interface chill/sand (W m^−2^ °C^−1^)	Perfect contact
Graphite chill	Density (kg m^−3^)	2250.0
	Heat capacity (J kg^−1^ °C^−1^)	710.0
	Thermal conductivity (W m^−1^ °C^−1^)	140.0
	Mesh size (mm^3^)	4 × 4 × 4
Sand mold	Density (kg m^−3^)	1520.0
	Heat capacity (J kg^−1^ °C^−1^)	1070.0
	Thermal conductivity (W m^−1^ °C^−1^)	1.0
	Mesh size (mm^3^)	4 × 4 × 4

**Table 3:** Experimentally measured concentrations of Pb (wt.%) at various locations of the B–B plane divided into 6 × 6 grids.

GraphiteChill	6.152	6.228	6.320	6.098	5.854	5.989
	6.193	6.202	6.228	6.273	5.723	5.535
	6.089	6.124	6.168	6.150	6.170	5.259
	6.164	6.156	6.249	6.033	5.505	5.590
	6.058	6.023	6.091	6.062	5.196	5.575
	6.078	6.163	6.153	6.442	5.718	6.393

## Data Availability

The data presented in this study are available on request from the corresponding author.
